# Modeling County-Level Rare Disease Prevalence Using Bayesian Hierarchical Sampling Weighted Zero-Inflated Regression^✩^

**DOI:** 10.6339/22-JDS1049

**Published:** 2023-01

**Authors:** HUI XIE, DEBORAH B. ROLKA, LAWRENCE E. BARKER

**Affiliations:** 1 Centers for Disease Control and Prevention, National Center for Chronic Disease Prevention and Health Promotion, Division of Diabetes Translation, Atlanta, Georgia, USA; 2 Centers for Disease Control and Prevention, National Center for Chronic Disease Prevention and Health Promotion, Office of the Director, Atlanta, Georgia, USA (retired)

**Keywords:** excess zeros, incidence, PLOW, power prior, small area estimate

## Abstract

Estimates of county-level disease prevalence have a variety of applications. Such estimation is often done via model-based small-area estimation using survey data. However, for conditions with low prevalence (i.e., rare diseases or newly diagnosed diseases), counties with a high fraction of zero counts in surveys are common. They are often more common than the model used would lead one to expect; such zeros are called ‘excess zeros’. The excess zeros can be structural (there are no cases to find) or sampling (there are cases, but none were selected for sampling). These issues are often addressed by combining multiple years of data. However, this approach can obscure trends in annual estimates and prevent estimates from being timely. Using single-year survey data, we proposed a Bayesian weighted Binomial Zero-inflated (BBZ) model to estimate county-level rare diseases prevalence. The BBZ model accounts for excess zero counts, the sampling weights and uses a power prior. We evaluated BBZ with American Community Survey results and simulated data. We showed that BBZ yielded less bias and smaller variance than estimates based on the binomial distribution, a common approach to this problem. Since BBZ uses only a single year of survey data, BBZ produces more timely county-level incidence estimates. These timely estimates help pinpoint the special areas of county-level needs and help medical researchers and public health practitioners promptly evaluate rare diseases trends and associations with other health conditions.

## 1 Introduction

Disease or condition prevalence data are often gathered at the state (e.g., Behavioral Risk Factor Surveillance System (BRFSS)) or national (e.g., National Health Information Survey) level. However, estimates at a finer geographical scale, such as county, are often needed. In these cases, small area estimation (SAE) gives us a way forward. Model-based SAE can deliver more precise estimates of the parameters of interest than direct methods (Sugasawa and Kubokawa, 2020; Ghosh and Rao, 1994). There are two main types of model-based approaches: frequentist and Bayesian. Although both are used in SAE, the latter has several advantages (Trevisani and Torelli, 2017; Best et al., 2019). These include increased flexibility in dealing with complex models and the ability to accommodate many sources of uncertainty, which can be integrated into posterior distribution.

According to the Orphan Drug Act of 1983, a rare disease is defined as a condition affecting fewer than 200,000 people. The demand for estimates of county-level rare disease prevalence has increased dramatically over the last few years (Auvin et al., 2018; Liu et al., 2017; Bendewald et al., 2010; Thompson et al., 2007). Such estimates allow researchers and policymakers to better understand disease trends and to better target prevention efforts. There are two main challenges to estimate county-level rare disease prevalence: (1) surveys conducted at a larger scale than county often have very few respondents in each county (some counties may have no survey data whatsoever) and (2) because the disease of interest is rare, few are likely to be observed in each county. These issues are often addressed by combining multiple years of data. However, this approach can obscure trends in annual estimates and prevent estimates from being timely. Even with combined years, many counties may still have no observed cases.

Bayesian hierarchical regression (BHR), a type of model-based estimation, plays a vital role in SAE. Erciulescu et al. (2019) proposed BHR to estimate county-level acreage and crop production by incorporating remote sensing data, weather data and planted acreage administration data as auxiliary variables. Similarly, Alexander et al. (2017) presented a Bayesian hierarchical Poisson regression to estimate county-level mortality rates with three hierarchies by borrowing variances across all counties. Extensions of BRH have been made by introducing spatio-temporal variations (Khana et al., 2018; Ayubi et al., 2018) and sampling weights adjustment (Chen et al., 2015; Vandendijck et al., 2016). However, BHR models implemented with a binomial or Poisson distribution can only be rough approximations because data are often overdispersed (e.g., more zeroes than the parametric model accounts for) (Millar, 2009). Not accounting for overdispersion causes the estimated variances of parameter estimates to be negatively biased (Lee et al., 2012). Several distributions, such as negative binomial, zero-inflated Poisson, and zero-inflated beta-binomial, have been used in cases where there are more zeros than a binomial or Poisson model would allow (Dai et al., 2018; Hu et al., 2018; Pourhoseingholi et al., 2018).

Recently, a new method (Xie et al., 2020) of SAE was proposed: Power prior LOg-Weights estimates (PLOW). PLOW involves a BHR model with power prior distribution and introduces adjusted sample weights on account of the design mechanism. However, PLOW does not account for there being more zero counts than one would see under a binomial model. Here, we expand PLOW by incorporating a zero-inflated binomial distribution to estimate the county-level prevalence of a rare condition. We call this approach Bayesian Weighted Binomial Zero-inflated distribution (BBZ). In short, BBZ extends PLOW by implementing zero-inflated binomial distribution on account of excess zero counts (overdispersion).

As an example, we use BBZ to estimate the county-level prevalence of young adults (18 to 35 years old) who have self-care difficulty (DDRS) (having trouble with dressing, bathing or getting around inside the home because of his/her physical, mental or emotional condition), using BRFSS data. The results are validated with American Community Survey (ACS) 1-year reports.

## 2 Motivating Study

The BRFSS is a state-level annual telephone survey study conducted by the Centers for Disease Control and Prevention among noninstitutionalized adults aged 18 years or older in the United States and some territories. BRFSS 2019 was the most recent data available to the public at the time of this study. In 2019, the median response rate of all states was 45.9%. The total sample size was about 450,000. As there are 3142 county or county-equivalents in the United States, many counties had very small sample sizes. As a state-level survey, the surveyed samples assigned to each county is relatively small; two hundred and thirty-four counties (~7%) have no data. Indeed, since no BRFSS 2019 data were collected from New Jersey, all of the state’s 21 counties had no data. Thus, these left only 2908 counties with available data in 2019.

The ACS, conducted by the Census Department to track nationwide health, jobs and occupations, educational attainments, housings and other topics, uses four modes: internet, mail, telephone, and personal visit (Gettens et al., 2015). In 2019, the response rate was 86.0%. The sample size is around 3.5 million each year, which is 8 to 9 times larger than BRFSS. The ACS releases two versions of county-level reports every year: ACS 1-year and ACS 5-year. ACS reports 5-year data annually for all counties by aggregating five years of survey data, while ACS 1-year reports are based on single-year survey data only for the large-size counties (population size *>* 65,000) (~27%). Therefore, we validate our estimates using ACS county-level 1-year results.

DDRS is rare in young adults (those aged between 18 and 35 years). According to the 2019 ACS 5-year report for 3121 counties, the DDRS prevalence rate in young adults was 0.86%, 13.49% in those aged 75+ years, and 2.71% in the entire population. We use DDRS in young adults as our example rare disease because both ACS and BRFSS have been collecting DDRS data since 2013, and both ACS and BRFSS ask the same question, “Do you have difficulty dressing or bathing?”.

## 3 Statistical Models

### 3.1 Bayesian Hierarchical Regression (BHR) Model

To address the problem of small sample sizes, we apply BHR by borrowing “strength” across whole counties and states and auxiliary variables. We cross-classify respondents into three age groups (18 to 24, 25 to 29 and 30 to 35 years old), two sex groups (male and female) and two race groups (white and non-white; sample sizes make narrower classification impractical), which resulted in 12 clusters (e.g., cluster of white males aged 18 to 24 years old). Let $y_{ij}$ be count of young adult DDRS cases in county i and cluster j (i = 1, 2,..., 3142, j = 1, 2, ..., 12). For ${\text{k}}^{th}$ respondent, in particular, $y_{ijk}$*=* 1 denotes DDRS case and $y_{ijk}$ *=* 0 otherwise. We assume $y_{ij}$ followed a binomial distribution. The models can be defined as a pair of equations (Barker et al., 2013):

(3.1) $$y_{ij}=\sum_{k=1}y_{ijk}\;\text{and}\;y_{ij}|p_{ij},n_{ij}∼\text{Binomial}\left(p_{ij},n_{ij}\right)$$

(3.2) $$\log\left(\frac{p_{ij}}{1-p_{ij}}\right)=X\beta_{j}+Z\gamma_{j}+\mu_{ij}+v_{s(i)j}$$

where $p_{ij}$ and $n_{ij}$ are probability of cases and sample sizes in county i and cluster j, respectively. X is the vector of 12 clusters and Z is vector of auxiliary variables (i.e., education level, poverty rate, etc.). The $\mu_{ij}$ and $v_{s(i)j}$ are random effects of ${\text{i}}^{th}$ county and ${\text{s}}^{th}$ state in cluster j, respectively. We assume $\mu_{ij}$ and $v_{s(i)j}$ are independent. The posterior distribution of $p_{ij}$ given $y_{ij}$:

(3.3) $$f\left(p_{ij}|y_{ij}\right)\propto L\left(y_{ij}|p_{ij}\right)\pi \left(p_{ij}\right)$$

where f(.∣.)*, L(.|.)*, and π(.∣.) are denoted as the posterior distribution, the likelihood function, and the prior distribution of $p_{ij}$ given $y_{ij}$, respectively, and hereafter.

### 3.2 PLOW: Power Prior Sampling Log-Weight Adjustment Method

Typically, BHR conditions on the samples and the parameters of interest. That is, the sampling design mechanisms are not used (Pfeffermann, 2013). In Equation 3.1, $y_{ij}$ is the sum of case counts in county-level which is independent of the sampling design and weights. Studies (Kish and Frankel, 1974; Hansen et al., 1983) show that not accounting for sampling weights can cause both biased estimates and large variance of estimates. However, inappropriately incorporating sampling weights to BHR can also result in poor model fit. For example, extremely large or small sampling weights can result in estimates with a large variance. Second, the likelihood part weakly influences the posterior distribution when there are few or no observed data. In other words, the estimates of parameters of interest for those counties are primarily determined by the prior distribution. If non-informative priors (a common choice) are used, the results are “diffuse”.

To solve these problems, we adapt PLOW in this study. Firstly, we calculate the “effective” case counts by introducing sampling weights:

(3.4) $$y_{ij}^{e}=\frac{\sum_{k=1}{\left(\log\left(w_{ijk}\right)\right)}^{T}y_{ijk}}{\sum_{k=1}{\left(\log\left(w_{ijk}\right)\right)}^{T}}y_{ij}$$

where, $y_{ij}^{e}$ is the “effective” case counts in ${\text{i}}^{th}$ county and ${\text{j}}^{th}$ cluster. The $w_{ijk}$ is sampling weight corresponding to ${\text{k}}^{th}$ respondent. Here, we use T as an index of transformation in the range 0 to 1, in other words, T∈[0.1], is a tuning parameter (Xie et al., 2020); in Tukey’s ladder of transformations, a logarithmic transformation corresponds to an asymptotically zero exponent. In particular, T=0 corresponds to the unweighted adjustment while = 1 to the fully-weighted adjustment. Using “effective” counts $y_{ij}^{e}$, Equation 3.1 is modified as:

$$y_{ij}^{e}|p_{ij}∼\text{Binomial}\left(p_{ij},n_{ij}\right)$$

Secondly, assuming historical data are available, we construct a power prior (Chen et al., 2000). This approach is a compromise between non-informative priors and historical data. To estimate the prevalence of young adult DDRS in 2019, we use 2017 and 2018 BRFSS data as historical data. Both BRFSS 2017 and BRFSS 2018 had the same questions on DDRS and survey designs as BRFSS 2019.

Let $Y_{0ij}$ and $p_{0ij}$ be the counts and probability of historical cases in county i and cluster j, respectively. The posterior distribution of the power prior is defined as:

$$f\left(p_{0ij}|Y_{0ij},\alpha_{0}\right)\propto L{\left(Y_{0ij}|p_{0ij}\right)}^{\alpha_{0}}\pi \left(p_{0ij}\right)$$

where, L(.∣.) is likelihood function; $\alpha_{0}\in (0,1)$ is an empirically determined power parameter which controls the “strength” borrowing from the historical data.

By introducing of the sampling weight and power prior, the posterior distribution of $p_{ij}$ (3.3) is:

(3.5) $$f\left(p_{ij}|y_{ij}^{e},Y_{0ij},\alpha_{0}\right)\propto L\left(y_{ij}^{e}|p_{ij}\right)\pi \left(p_{0ij}|Y_{0ij},\alpha_{0}\right)$$

$$=L\left(y_{ij}^{e}|p_{ij}\right)L{\left(Y_{0ij}|p_{0ij}\right)}^{\alpha_{0}}\pi \left(p_{0ij}\right)$$

We call $L\left(y_{ij}^{e}|p_{ij}\right)L{\left(Y_{0ij}|p_{0ij}\right)}^{\alpha_{0}}$ the “power” likelihood function. With the proper conjugate beta distribution of power prior π(.∣.)∼beta(α,β), the posterior distribution of $p_{ij}$ follows a beta-binomial distribution:

(3.6) $$f\left(p_{ij}|y_{ij}^{e},Y_{0ij},\alpha_{0}\right)\propto p_{ij}^{y_{ij}^{e}+Y_{0ij}\times \alpha_{0}+\alpha_{i}-1}{\left(1-p_{0ij}\right)}^{y_{ij}^{e}+Y_{0ij}\times \alpha_{0}+\beta_{i}-1}$$

### 3.3 Bayesian Hierarchical Weighted Binomial Zero-Inflated Regression (BBZ)

Observed data often have excess zeros, compared to models implemented with standard distributions, such as binomial or Poisson. The excess zeros can be structural (there are no cases to find) or sampling (there are cases, but none were selected for the sample). Here, we apply the zero-inflated binomial distribution to process structure zeros, sampling zeros and positive counts, simultaneously Letting $\omega_{i}$ be the probability that an observation is zero in ${\text{i}}^{th}$ county, the probability density function of the zero-inflated binomial is:

$$f\left(y_{ij}^{e};\omega_{i},p_{ij},n_{ij}\right)=\{\begin{array}{ll} \omega_{i}+\left(1-\omega_{i}\right)f\left(y_{ij}^{e};p_{ij},n_{ij}\right), & y_{ij}^{e}=0\\ \left(1-\omega_{i}\right)f\left(y_{ij}^{e};p_{ij},n_{ij}\right), & y_{ij}^{e}>0 \end{array}$$

where the binomial function $f\left(y_{ij}^{e};p_{ij},n_{ij}\right)$ is defined as:

(3.7) $$f\left(y_{ij}^{e};p_{ij},n_{ij}\right)=\left(\begin{array}{c} n_{ij}\\ y_{ij}^{e} \end{array}\right)p_{ij}^{y_{ij}^{e}}{\left(1-p_{ij}\right)}^{\left(n_{ij}-y_{ij}^{e}\right)}$$

Finally, combined with three features of sampling weight, power prior and zero-inflated distribution, the posterior distribution of $p_{ij}$ (3.5) is updated as:

(3.8) $$f\left(p_{ij}|y_{ij}^{e},Y_{0ij},\alpha_{0},\omega_{i}\right)\propto L\left(y_{ij>0}^{e}|p_{ij},\omega_{ij}\right)\times L\left(y_{ij=0}^{e}|p_{ij},\omega_{i}\right)$$

$$\times L\left(Y_{0ij>0}|p_{0ij},\alpha_{0},\omega_{i}\right)L\left(Y_{0ij=0}|p_{0ij},\alpha_{0},\omega_{i}\right)\pi \left(p_{0ij}\right)$$

where $\pi \left(p_{0ij}\right)$ is non-informative initial prior for power prior π(.∣.), which is assigned as $\pi \left(p_{0ij}\right)\propto$ Normal (0, var = 10^6^). And $\omega_{i}$ has same non-informative prior as $\pi \left(p_{0ij}\right)$.

Once $p_{ij}$ is established, it is straightforward to calculate the estimated prevalence of DDRS in count i, $p_{i}$, as:

$$p_{i}=\frac{\sum_{j=1}^{12}p_{ij}N_{ij}}{\sum_{j=1}^{12}N_{ij}}$$

where $N_{ij}$ is county-level young adult population projections in county i and cluster j derived from US Census Bureau county-level population projections.

### 3.4 Model Validations

Four BHR models are evaluated. These models assume: binomial distribution (BHBI); zero-inflated binomial distribution (BZBI); PLOW (BPLW); and our new approach, BBZ. BHBI is a default model that fits binary counts without considering any specific datafeature; BZBI takes care of excess zeros; BPLW includes sampling weight and power prior; BBZ takes into account these elements: survey design, prior distribution and zero-inflated. Each model is applied to estimate the county-level DDRS prevalence in young adults using BRFSS 2019. Meanwhile, we check the impact of different levels of “zero” counts on the model performance using simulation data at the county-level.

ACS reports are often treated as “gold” standard because ACS is a survey large enough to provide direct estimates for many counties. The ACS releases single-year disability data for the 835 large-size counties. For model validation purposes, we selected 228 large-size counties with a population of young adults (aged from 18 to 35 years old) at least 65,000.

The Root Mean Square Error (RMSE) is a common method of assessing model performance:

$$RMSE=\sqrt{\frac{\sum_{i=1}^{m}{\left(p_{i}-p_{ACSi}\right)}^{2}}{m}}$$

where, m is the number of selected counties, $p_{i}$ is model-based county-level estimate and $p_{ACSi}$ is the ACS county-level 1-year report. The other criterion is Mean Bias Error (MBE). MBE measures the deviation of estimates ($p_{i}$) from the best approximation to the actual values ($p_{ACSi}$):

$$MBE=\frac{\sum_{i=1}^{m}\left(p_{i}-p_{ACSi}\right)}{m}$$

The Deviance Information Criterion (DIC) is particularly useful to check the goodness-of-fit of Bayesian models. DIC is calculated as (Spiegelhalter et al., 2002; Shriner and Yi, 2009):

$$DIC=2\bar{D}\left(y,p_{i}\right)-D\left(y,\bar{p_{i}}\right)$$

where, $\bar{D}\left(y,p_{i}\right)$ is posterior mean deviation and $D\left(y,\bar{p_{i}}\right)$ is the deviation at posterior mean $\bar{p_{i}}$, respectively. For each of these three indices, smaller values indicate better model performance.

All analyses were performed using SAS (version 9.4). PROC MCMC implemented with Monte Carlo Markov chain (MCMC) was applied to draw the samples corresponding the posterior distributions.

## 4 Results

### 4.1 Using BRFSS data

**Figure 1:**
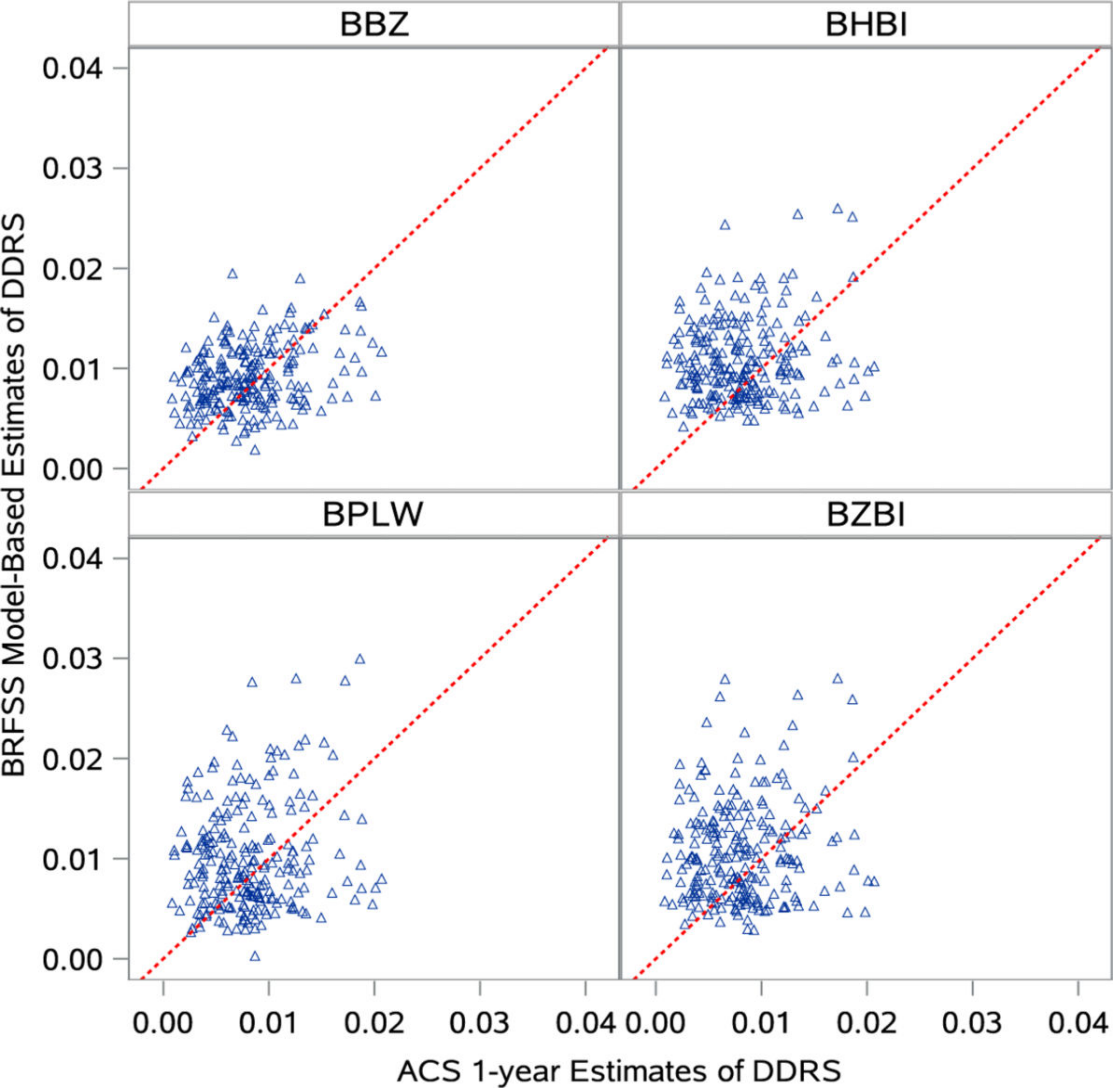
Scatter plots of agreements between model-based estimates with ACS 1-year reports of self-care difficulty (DDRS) in 2019 (BHBI: Bayesian hierarchical binomial regression; BZBI: Bayesian hierarchical zero-inflated binomial regression; BPLW: Bayesian hierarchical binomial regression with PLOW (Power prior sampling LOg-Weight Adjustment); BBZ: Bayesian hierarchical weighted zero-inflated binomial regression). Each spot represents one county.

Two hundred and twenty-eight counties with young adults population size greater than 60000 were selected as “motive” samples for validation. The four models described above were applied to estimate the county-level prevalence of young adults with DDRS in 2019 using BRFSS 2019. Figure 1 shows the 2019 agreement between BRFSS model-based estimates and ACS 1-year reports of county-level DDRS. The reference line denoted what would happen if model-based estimates and standard references were identical. Among the four models, the BBZ estimates consistently produced the smallest RMSE. More simulated studies results to test the performance of these four models, based on 2015 and 2016 BRFSS data, are presented in Supplementary Materials (Figures 4 & 5).

Table 1 showed BBZ had a 31.4% and 46.8% smaller RMSE and MBE than BPLW due to the binomial zero-inflated distribution. BBZ was about 25.4% and 62.2% smaller RMSE and MBE than BZBI due to its use of the PLOW method. BBZ had the smallest DIC which indicated the best model fit. BHBI had the highest RMSE and DIC amongst the four models.

**Figure 2:**
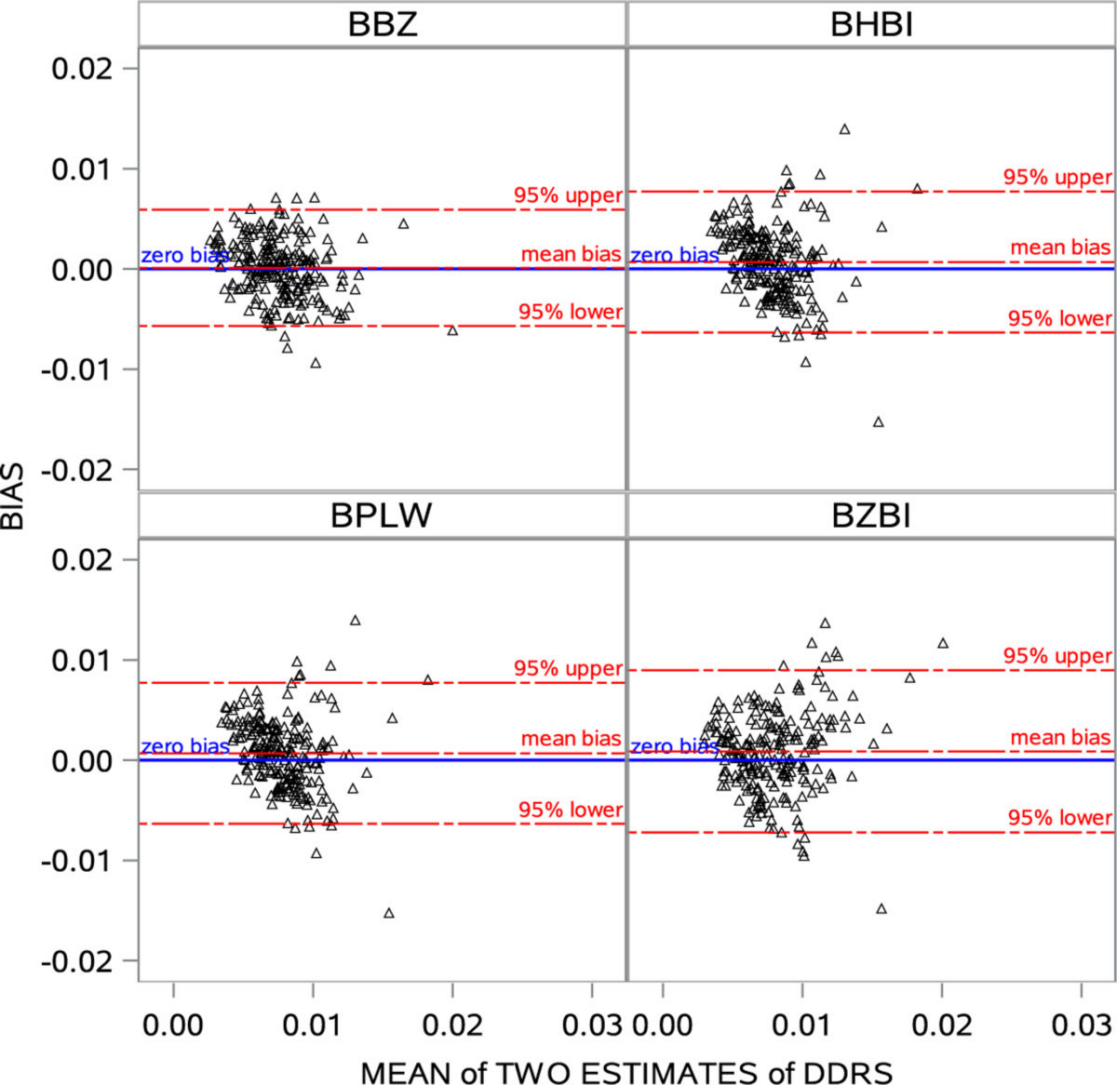
Bland-Altman plots and analysis of four models using BRFSS 2019 (BHBI: Bayesian hierarchical binomial regression; BZBI: Bayesian hierarchical zero-inflated binomial regression; BPLW: Bayesian hierarchical binomial regression with PLOW (Power prior sampling LOg-Weight Adjustment); BBZ: Bayesian hierarchical weighted zero-inflated binomial regression).

The Bland-Altman plot is a visualization method to assess bias patterns (Bland and Altman, 1999). It plots the difference of two measures (bias) on the Y-axis against the average of the two measures (mean) at the X-axis and overlays reference lines, such as 95% upper (mean+1.96∗SD_mean_) and 95% lower (mean-1.96∗SD_mean_) limits in the same plot. Figure 2 presents the Bland-Altman plots of our four models using BRFSS 2019. The points were approximately equally distributed below and above the “zero bias” line, suggesting no systematic errors. However, plots of BHBI and BZBI presented “cone” shapes, in which points lie closer to the ‘zero bias’ line on the left and spread out as one moved to the right. This suggests that biases were proportional to the magnitude of measures. Furthermore, some points were far from the upper 95% limits lines, suggesting a right-tail skew. In the BPLW plot, a cluster of points suggested a “trend”, in which points tended to be overestimated for smaller values of the parameters of interest and underestimated for larger. We found no such patterns in the BBZ plot. Besides, BBZ had the narrowest 95% confidence interval (−5.7 × 10^−3^, 5.9 × 10^−3^).

**Figure 3:**
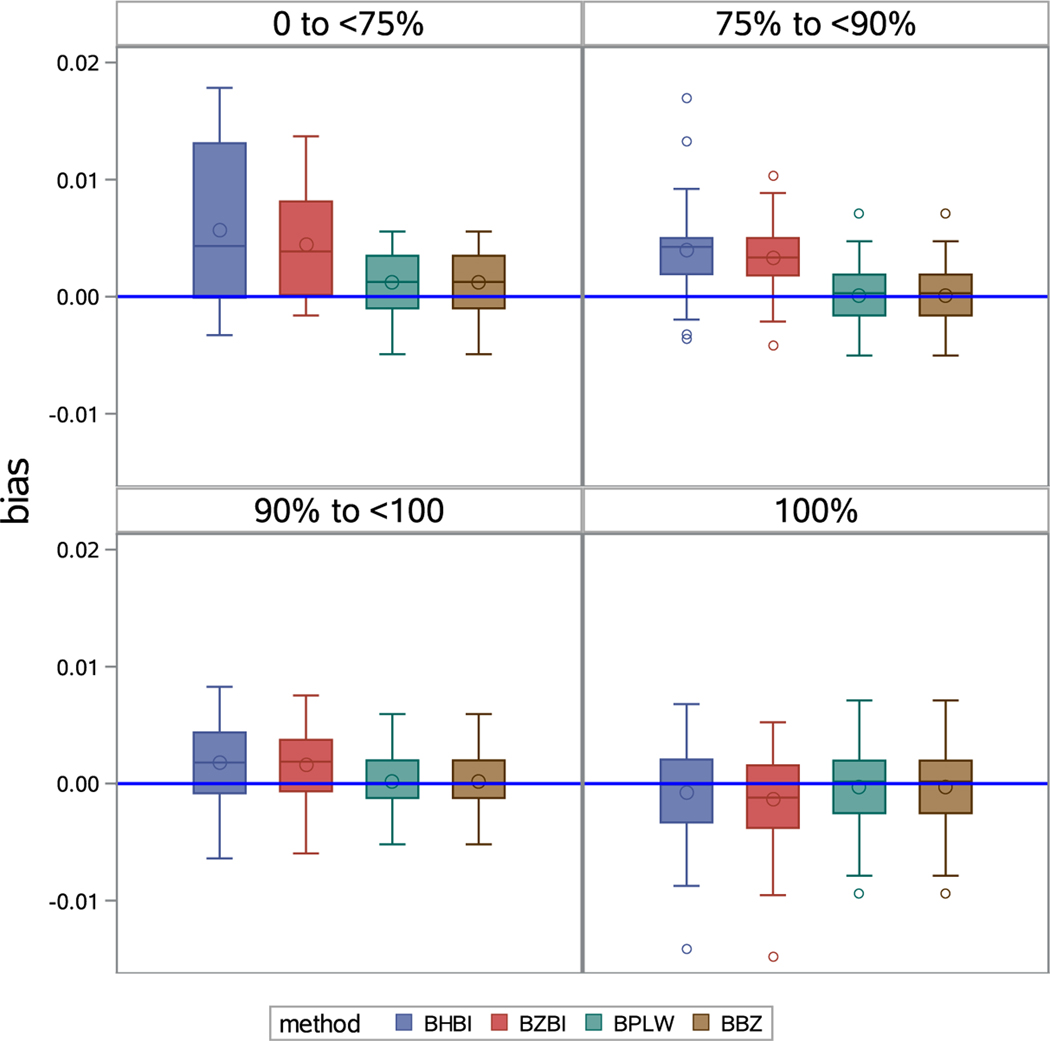
Distribution of bias at different “zero” counts levels; BHBI: Bayesian hierarchical binomial regression; BZBI: Bayesian hierarchical zero-inflated binomial regression; BPLW: Bayesian hierarchical binomial regression with PLOW (Power prior sampling LOg-Weight Adjustment); BBZ: Bayesian hierarchical weighted zero-inflated binomial regression.

We also test the bias at different levels of having “zero” counts. Based on BRFSS 2019 DDRS survey data, we classify all counties into one of four “zero” levels: 0 to *<*70%, 70% to *<*90%, 90% to *<*100%, and 100% (these categories are arbitrary but are considered ‘reasonable’). Figure 3 shows box and whisker plots for bias in the four “zero” levels. This figure suggests that bias varies by level of zeros less with BBZ than the other models. BHBI and BZBI are more likely to create positively biased results at levels “0 to *<*70%” and “70% to *<*90%”. BPLW varies widely, with positive bias in the “0 to *<*70%” level. 48.4% counties have no DDRS cases (100% zeroes). At this level, the plots show the four models perform roughly similarly.

### 4.2 Using “Pseudo-Counties” Data

We investigate the impact of the “zero” count levels more generally through simulation. First, we create 228 “pseudo-counties” by resampling from those “super-large” counties at 50%, 60%, 70%, 80%, 90% and 95% levels of “zero” counts of DDRS, respectively, using 2019 BRFSS county-level DDRS data. Then, we apply each of four models (BHBI, BZBI, BPLW and BBZ) to the “pseudo-counties”. The results are compared to the ACS 1-year reports (Table 2).

In every case, BBZ outperforms to the other models, with lower RMSE and DIC values from 90% down to 50% “zero” levels. In terms of RMSE, the BZBI has similar performance with BHBI at levels of 80% and above. At the 95% level, RMSE values for the four models are similar. This is consistent with Figure 3.

## 5 Discussion

We developed a new approach, BBZ, to estimate county-level rare disease prevalence. BBZ features: a Bayesian hierarchical model, the PLOW method, and a zero-inflated distribution. These features allow us to address two challenges that are common in SAE of the prevalence of rare diseases: (1)very small sample sizes or no data in some counties; (2)high volumes of “zero” counts. Traditionally, the zero-inflated or hurdle or truncated models (Weaver et al., 2015; Rose et al., 2006) have been employed to deal with excess “zero” counts. Our results show that the BHBI can decrease variance but result in positive bias. Other zero-inflated models were considered but yielded similar results. Positive bias probably arises from a failure to consider the sample design and sampling weights associated with the data. A previous study (Xie et al., 2020) showed that the use of PLOW could dramatically reduce bias and variance in SAE. However, the BPLW method is not designed to handle “over-dispersion” and zero-inflated. Furthermore, if a case is counted as zero, the corresponding sampling weight is not useful, because PLOW is only applied to the non-zero cases.

BBZ, which simultaneously integrates the PLOW method and zero-inflated distribution, performs well and has the lowest bias. Findings derived from both empirical and simulation data demonstrate that BBZ provides the best performance at any “zero” level. In addition, BBZ uses “historical” data through the use of a power prior distribution; this tends to improve the accuracy, especially for counties with very small/zero sample sizes. Some studies (Khan et al., 2018; Gibbs et al., 2020; Oleson et al., 2008; Vahedi et al., 2021) suggested incorporating the spatial random effect by borrowing strength from space can improve model fit and estimate accuracy. The topic is slightly out of the scope of this study, we may explore the spatial random effect in the BBZ and other BHR models in the future study.

In the study of rare diseases, it is common for the sample to have no cases in some counties. Even among the 228 large-sized counties used as motivating samples in this study, 82 had no DDRS young adult cases in the 2019 BRFSS. If the counts are 100% “zero”, the data are not binary. We showed that all models performed similarly – very low mean (close to “0”) but high variance for the competing models considered. This could be explained by the facts that all “zero” counts are fit by the degenerate distribution (Bhattacharya et al., 2008; Tang et al., 2015) and the variances come from models borrowing “strength” directly from other counties and states (Porter et al., 2015; Rao and Molina, 2015).

Although BBZ is used to estimate county-level young adult DDRS prevalence in this study, no unique properties of DDRS were used. Thus, BBZ can be used for county-level studies of any rare condition, such as new cases of diabetes. Diabetes incidence is defined as newly-diagnosed disease cases; the annual rate is fairly low. For example, a CDC national survey () estimated this rate as 0.67% among U.S. adults aged 18 years or older in 2018. Since relatively few survey respondents with diabetes are new cases, the resulting county-level case counts are very small with many excess zero counts. BBZ is ideal for estimating county-level diabetes incidence with small sample sizes as it uses both zero-inflated distribution and PLOW.

Many methods historically used to estimate county level incidence or prevalence of diseases combine multiple years of data (Rossen et al., 2018; Cadwell et al., 2010), which hampers timeliness and obscures secular trends. Since BBZ uses only a single year of survey data, BBZ produces more timely county-level incidence estimates. These timely estimates make it possible for the researchers to promptly investigate disease trends and for policymakers to better target control and prevention efforts.

## Supplementary Material

Appendix 1. Figure 4: Agreement between BRFSS model-based estimates and ACS 1-year reports of county-level DDRS based on 225 selected counties in 2015. The reference line denotes if model-based estimates and standard references (e.g., ACS 1-year report) were identical. Among the four models (BHBI, BZBI, BPLW and BBZ), estimates of BHBI and BZBI present both large variances and bias; Most counties have a positive estimated bias. Estimates of BBZ tend to stay closer to the reference line with least bias and variance. These results are matched with those in 2019.
Figure 5: Agreement between BRFSS model-based estimates and ACS 1-year reports of county-level DDRS based on 225 selected counties in 2016. The reference line denotes if model-based estimates and standard references (e.g., ACS 1-year report) were identical. Among the four models (BHBI, BZBI, BPLW and BBZ), estimates of BHBI and BZBI present both large variances and bias; Most counties have a positive estimated bias. Estimates of BBZ tend to stay closer to the reference line with least bias and variance. These results are matched with those in 2019.

## Figures and Tables

**Table 1:** The values of Deviance Information Criterion (DIC) and root mean square error (RMSE) of Four Models (BHBI, BZBI, BPLW and BBZ) using Behavioral Risk Factor Surveillance System (BRFSS) 2019 data, respectively (BHBI: Bayesian hierarchical binomial regression; BZBI: Bayesian hierarchical zero-inflated binomial regression; BPLW: Bayesian hierarchical binomial regression with PLOW (Power prior sampling LOg-Weight Adjustment); BBZ: Bayesian hierarchical weighted zero-inflated binomial regression). Smaller values of DIC, RMSE and MBE indicate better fit.

Model	BHBI	BZBI	BPLW	BBZ

RMSE (×10^−3^)	6.73	6.02	6.55	4.49
MBE (×10^−3^)	2.60	2.70	1.92	1.02
DIC	3436.29	3480.38	3341.3	2775.16

**Table 2:** The impact of different “zero” counts levels on the model performance using “pseudo-counties”.

	95% “zero”		90% “zero”		80% “zero”		70% “zero”		60% “zero”		50% “zero”	
	RMSE (×10^−3^)	DIC	RMSE (×10^−3^)	DIC	RMSE (×10^−3^)	DIC	RMSE (×10^−3^)	DIC	RMSE (×10^−3^)	DIC	RMSE (×10^−3^)	DIC

BHBI	4.9	1861	7.1	3498	15.8	6296	23.3	8862	28.8	11204	36.9	13124
BZBI	4.6	1881	6.6	3546	15.7	6389	19.4	9059	25.8	11431	32.2	13375
BPLW	4.2	1906	4.7	3354	10.9	5720	14.1	8019	20.1	10069	26.1	11823
BBZ	4.0	1311	3.3	1945	8.8	2855	12.9	3562	16.0	4337	21.5	4972
