# Supplementary material for: Modeling County-Level Rare Disease Prevalence Using Bayesian Hierarchical Sampling Weighted Zero-Inflated Regression
Source: J Data Sci. Author manuscript; Available in PMC 2024 May 24. (PMC11119276; doi:10.6339/22-JDS1049)
Supplement: Appendix 1. — Figure 4: Agreement between BRFSS model-based estimates and ACS 1-year reports of county-level DDRS based on 225 selected counties in 2015. The reference line denotes if model-based estimates and standard references (e.g., ACS 1-year report) were identical. Among the four models (BHBI, BZBI, BPLW and BBZ), estimates of BHBI and BZBI present both large variances and bias; Most counties have a positive estimated bias. Estimates of BBZ tend to stay closer to the reference line with least bias and variance. These results are matched with those in 2019. Figure 5: Agreement between BRFSS model-based estimates and ACS 1-year reports of county-level DDRS based on 225 selected counties in 2016. The reference line denotes if model-based estimates and standard references (e.g., ACS 1-year report) were identical. Among the four models (BHBI, BZBI, BPLW and BBZ), estimates of BHBI and BZBI present both large variances and bias; Most counties have a positive estimated bias. Estimates of BBZ tend to stay closer to the reference line with least bias and variance. These results are matched with those in 2019. [file NIHMS1979449-supplement-Appendix_1_.docx]

APPENDIX

Figure 1 and 2 (suppl.) describes the 2015 and 2016, respectively, agreement between BRFSS model-based estimates and ACS 1-year reports of county-level DDRS based on 225 selected counties. The reference line denotes if model-based estimates and standard references (e.g., ACS 1-year report) were identical. Among the four models (BHBI, BZBI, BPLW and BBZ), estimates of BHBI and BZBI present both large variances and bias; Most counties have a positive estimated bias. Estimates of BBZ tend to stay closer to the reference line with least bias and variance. These results are matched with those in 2019.

| BRFSS Model-Based Estimates | 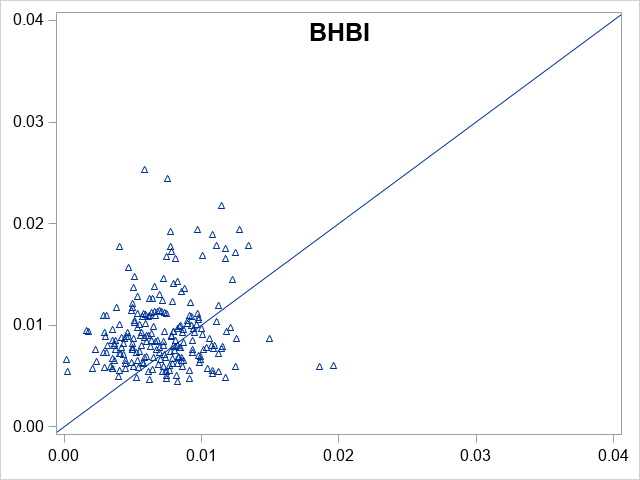 | 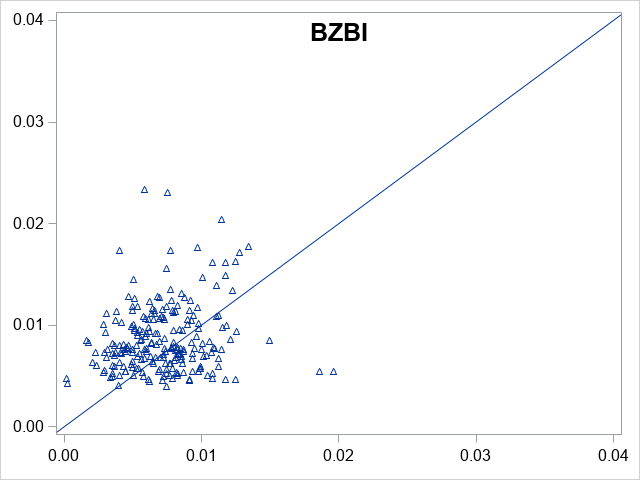 |
| --- | --- | --- |
| BRFSS Model-Based Estimates | 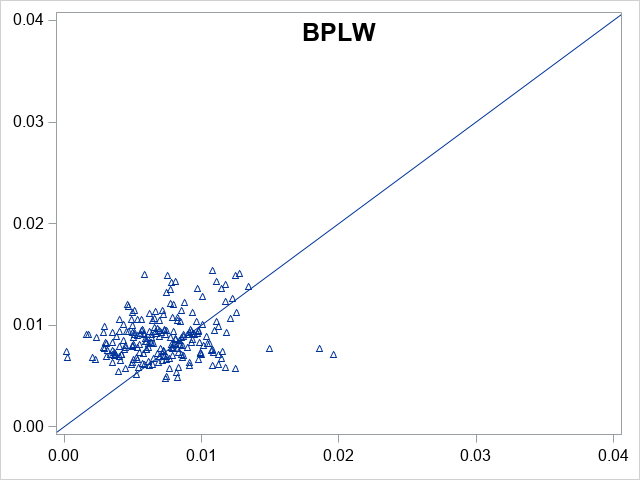 | 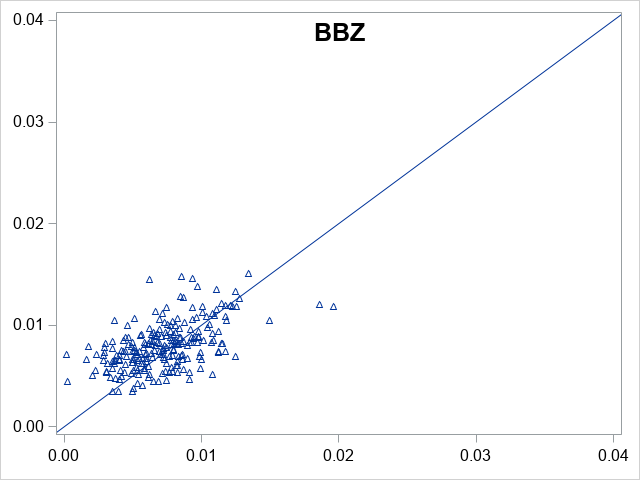 |
|  | ACS 1-year Estimates | ACS 1-year Estimates |

Figure 1(suppl). Scatter plots of agreements between model-based estimates with ACS 1-year reports of DDRS in 2015 (BHBI: Bayesian hierarchical binomial regression; BZBI: Bayesian hierarchical zero-inflated binomial regression; BPLW: Bayesian hierarchical binomial regression with PLOW (Power prior sampling LOg-Weight Adjustment); BBZ: Bayesian hierarchical weighted zero-inflated binomial regression)

| BRFSS Model-Based Estimates | 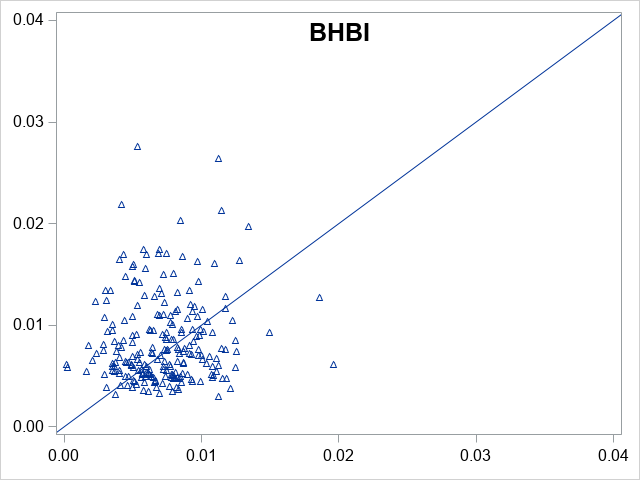 | 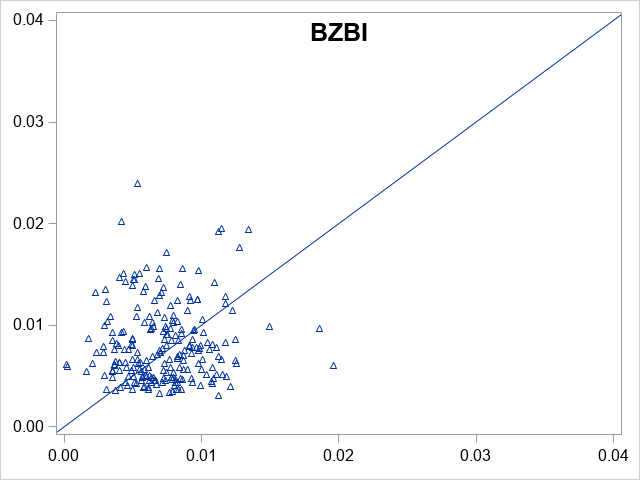 |
| --- | --- | --- |
| BRFSS Model-Based Estimates | 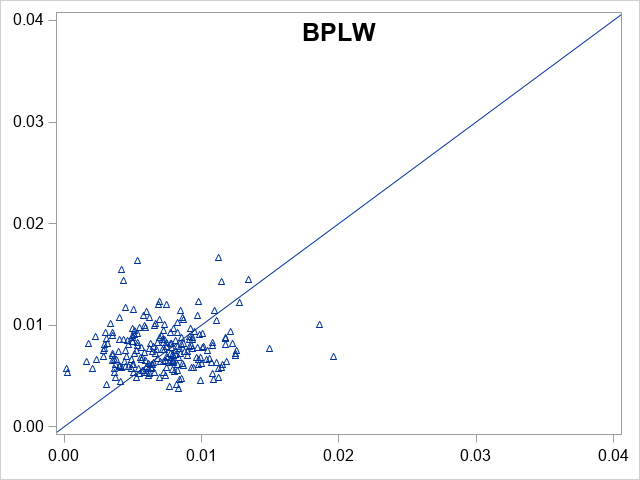 | 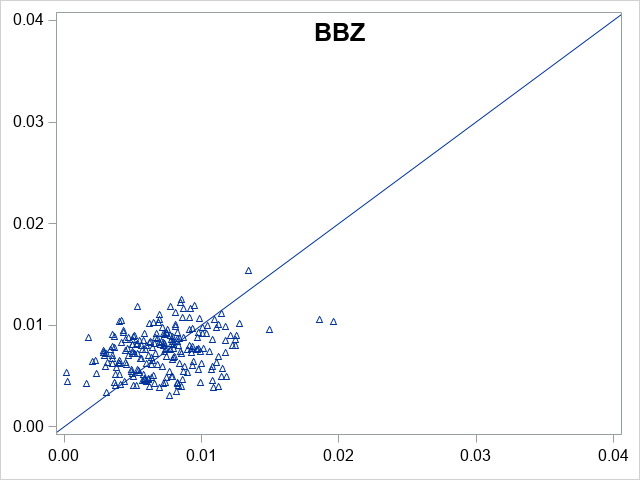 |
|  | ACS 1-year Estimates | ACS 1-year Estimates |

Figure 2 (suppl). Scatter plots of agreements between model-based estimates with ACS 1-year reports of DDRS in 2016 (BHBI: Bayesian hierarchical binomial regression; BZBI: Bayesian hierarchical zero-inflated binomial regression; BPLW: Bayesian hierarchical binomial regression with PLOW (Power prior sampling LOg-Weight Adjustment); BBZ: Bayesian hierarchical weighted zero-inflated binomial regression)
